# A comprehensive molecular analysis of 113 primary ovarian clear cell carcinomas reveals common therapeutically significant aberrations

**DOI:** 10.1186/s13000-023-01358-0

**Published:** 2023-06-12

**Authors:** Ivana Stružinská, Nikola Hájková, Jan Hojný, Eva Krkavcová, Romana Michálková, Jiří Dvořák, Kristýna Němejcová, Radoslav Matěj, Jan Laco, Jana Drozenová, Pavel Fabian, Jitka Hausnerová, Gábor Méhes, Petr Škapa, Marián Švajdler, David Cibula, Filip Frühauf, Michaela Kendall Bártů, Pavel Dundr

**Affiliations:** 1 Department of Pathology, First Faculty of Medicine, Charles University and General University Hospital in Prague, Prague, Czech Republic; 2 Department of Pathology, Faculty of Medicine, Charles University, University Hospital Kralovske Vinohrady, Prague, 3rd Czech Republic; 3 Department of Pathology and Molecular Medicine, Third Faculty of Medicine, Charles University, Thomayer University Hospital, Prague, Czech Republic; 4 The Fingerland Department of Pathology, Faculty of Medicine in Hradec Kralove, Charles University, University Hospital Hradec Kralove, Prague, Czech Republic; 5 Department of Oncological Pathology, Masaryk Memorial Cancer Institute, Brno, Czech Republic; 6 Department of Pathology, University Hospital Brno and Medical Faculty, Masaryk University, Brno, Czech Republic; 7 Department of Pathology, Faculty of Medicine, University of Debrecen, Debrecen, 4032 Hungary; 8 Department of Pathology and Molecular Medicine, Second Faculty of Medicine, Charles University and Motol University Hospital, Prague, Czech Republic; 9 Šikl’s Department of Pathology, The Faculty of Medicine, Faculty Hospital in Pilsen, Charles University, Pilsen, Czech Republic; 10 Gynecologic Oncology Center, Department of Obstetrics and Gynecology, First Faculty of Medicine, Charles University in Prague, General University Hospital in Prague, Prague, Czech Republic; 11 Department of Pathology, First Faculty of Medicine, Charles University and General University Hospital in Prague, Studničkova 2, Prague 2, 12800 Czech Republic

**Keywords:** Capture DNA NGS, RNA-Seq, Rare ovarian tumors, POLE mutation

## Abstract

**Background:**

Molecular aberrations occurring in primary ovarian clear cell carcinoma (OCCC) can be of diagnostic, predictive, and prognostic significance. However, a complex molecular study including genomic and transcriptomic analysis of large number of OCCC has been lacking.

**Methods:**

113 pathologically confirmed primary OCCCs were analyzed using capture DNA NGS (100 cases; 727 solid cancer related genes) and RNA-Seq (105 cases; 147 genes) in order to describe spectra and frequency of genomic and transcriptomic alterations, as well as their prognostic and predictive significance.

**Results:**

The most frequent mutations were detected in genes *ARID1A*, *PIK3CA*, *TERTp*, *KRAS*, *TP53*, *ATM*, *PPP2R1A, NF1*, *PTEN*, and *POLE* (51,47,27,18,13,10,7,6,6, and 4%, respectively). TMB-High cases were detected in 9% of cases. Cases with *POLE*^mut^ and/or MSI-High had better relapse-free survival. RNA-Seq revealed gene fusions in 14/105 (13%) cases, and heterogeneous expression pattern. The majority of gene fusions affected tyrosine kinase receptors (6/14; four of those were *MET* fusions) or DNA repair genes (2/14). Based on the mRNA expression pattern, a cluster of 12 OCCCs characterized by overexpression of tyrosine kinase receptors (TKRs) *AKT3*, *CTNNB1*, *DDR2*, *JAK2*, *KIT*, or *PDGFRA* (p < 0.00001) was identified.

**Conclusions:**

The current work has elucidated the complex genomic and transcriptomic molecular hallmarks of primary OCCCs. Our results confirmed the favorable outcomes of *POLE*^mut^ and MSI-High OCCC. Moreover, the molecular landscape of OCCC revealed several potential therapeutical targets. Molecular testing can provide the potential for targeted therapy in patients with recurrent or metastatic tumors.

**Supplementary Information:**

The online version contains supplementary material available at 10.1186/s13000-023-01358-0.

## Background

Ovarian clear cell carcinoma (OCCC) accounts for about 10% of ovarian carcinomas. Its occurrence is geographically different – in Europe and North America it has been estimated between 5 and 13%, whereas in Asia it makes up to 25% of all ovarian carcinomas [1, 2]. OCCC differs by pathological and molecular characteristics from other ovarian carcinomas, including endometrioid (EC), high grade serous (HGSC), low grade serous (LGSC), and mucinous carcinoma (MC).

Molecular aberrations occurring in OCCC can be of predictive and prognostic significance, however, a complex molecular study including genomic and transcriptomic analysis of large number of OCCC has been missing. Mutations occurring in OCCC have been analyzed in a limited number of studies [1, 3–11]. Some of these studies have, however, some limitations related mostly to the limited spectrum of genes analyzed, the sensitivity of the method (low coverage of WGS or WES), or a small sample set of analyzed cases. Nevertheless, it has been shown that mutations of *ARID1A*, *PIK3CA*, and *TERT* promoter are a common finding, followed by *KRAS*, *TP53*, *ATM*, and *PPP2R1A* mutations. The prognostic relevance of stratifying OCCC into different molecular subtypes has been suggested [3, 4, 12]. In one study, 421 OCCC were stratified into two main subgroups. One subgroup (“classic OCCC”) included tumors with *ARID1A* and other common mutations (such as *PIK3CA* and *TERT*) which represented about 83% of tumors. The second subgroup (“HGSC-like”) was characterized by the *TP53* mutation and showed an enriched expression of genes involved in extracellular matrix organization, mesenchymal differentiation, and immune-related pathways [3]. However, as admitted by the authors, the main weakness of this study is that some cases in the “HGSC-like” subgroup are probably true HGSC misclassified as OCCC. Another study of 55 OCCC suggested four different molecular subgroups: “PIK3CA”, “ARID1A”, “PIK3CA-ARID1A” and “Undetermined” [4]. Another possible approach is the stratification of OCCC into the Cancer Genome Atlas (TCGA)-based molecular subtypes used for endometrial carcinomas. Using this approach, *POLE* mutated (*POLE*^mut^) or mismatch repair deficient (MMR-D) cases have a better prognosis than p53 abnormal (p53abn) cases or cases with no specific molecular profile (NSMP) subgroup [12]. Better prognosis for MMR-D OCCC has been suggested also by the results of another study [13].

Concerning gene rearrangements, only two studies on a limited number of OCCC have focused on this topic to date [14, 15]. However, their results are problematic, given that in one of those studies (analyzing 4 OCCC) one tumor with detected fusion *CCNY*::*NRG4* actually represented a metastasis from primary uterine mixed clear cell and endometrioid carcinoma [14]. The second study analyzed 20 OCCCs in which three cases with several fusions were found, but the authors admitted the possibility of a methodological problem in analyzing the data and due to this their results are equivocal at best [15].

Concerning transcriptome analysis, only four studies performed expression profiling using RNA sequencing (RNA-Seq). One afore-mentioned large study combined a transcriptomic study with targeted DNA sequencing [3]. Another three studies included only a limited number of cases, namely 11, 19, and 6, respectively [16–18]. Two of them focused on the differences among ovarian carcinoma subtypes. The first study suggested the possible significance of expression analysis for differential diagnosis between OCCC and clear cell carcinoma of the uterus [16]. The second study examined these differences between OCCC, HGSC, and EC, while the third study mainly focused on the differences between OCCC and HGSC [17, 18]. Moreover, two other studies which used different methodological approaches (microarray analysis) included 37 and 8 OCCCs, respectively [19, 20].

Recently, epigenome profiling in OCCC has been performed in a collaborative study including 271 cases from ten study sites and genome-wide tumor DNA methylation profiling [21]. Their analyses supported the involvement of immune related pathways in OCCC and brought insight into epigenomic profiling. Furthermore, they revealed a higher level of chromosomal aneuploidy in OCCCs with *ARID1A*/*PIK3CA* mutation [21].

The primary goal of our study was to perform a comprehensive genomic and transcriptomic analysis of a well-defined sample set of 113 primary OCCCs with the aim to characterize these tumors with respect to the occurrence of molecular aberrations, as well as their prognostic and predictive value. Secondly, we focused on the possible stratification of our sample set into molecularly defined subgroups based on mutation and/or mRNA expression pattern.

## Methods

### Materials

The archives of the participating pathology departments were searched for cases originally diagnosed as OCCC. All cases were carefully reviewed by two pathologists (PD and MKB) and only cases meeting the strict morphological and immunohistochemical criteria were included into the study. The criteria included the morphology of clear cell carcinoma as defined elsewhere, associated with immunohistochemical profile compatible with the diagnosis, including positivity of PAX8 and at least one marker of “clear cell” differentiation (HNF1B, AMACR or napsin A) together with negativity of WT1 [22–24]. Finally, 120 OCCC cases were selected for DNA and RNA isolation. The clinicopathological characteristics of these cases are summarized in Table 1.

**Table 1:** Clinicopathological characteristics of 113 primary OCCCs.

Characteristics	OCCC (N = 113)	
Age (years)		
range	34–82	
mean/median	60.5/62	
**FIGO**	**(N = 104)**	
IA	38 (37%)	
IB	1 (1%)	
IC	36 (35%)	
II	8 (8%)	
III	20 (19%)	
IV	1 (1%)	
NA	9	
**T stage**	**(N = 105)**	
low (T1 + T2)	87 (83%)	
high (T3)	18 (17%)	
NA	8	
**N stage**	**(N = 101)**	
N0	48 (48%)	
N1	7 (7%)	
Nx	46 (46%)	
NA	12	
**M stage**	**(N = 101)**	
M0	6 (6%)	
M1	1 (1%)	
Mx	94 (93%)	
NA	12	
**Neoadjuvant therapy**	**(N = 81)**	
No	77	
Yes	4	
NA	32	
**Adjuvant therapy**	**(N = 95)**	
No	11 (12%)	
Yes	84 (88%)	
Chemotherapy	83 (99%)	
Chemotherapy and targeted treatment	1 (1%)	
NA	18	
**Lymphadenectomy**	**(N = 108)**	
No	53 (49%)	
Yes	55 (51%)	
NA	5	
**Recurrence**	**(N = 99)**	
No	68 (69%)	
Yes	31 (31%)	
Local (pelvic)	19 (61%)	
Distant	11 (35%)	
Combined	1 (3%)	
NA	14	
**Survival status (FU mean/median in months)**	**(N = 99)**	
NED (56/52)	58 (59%)	
AWD (44/29)	18 (18%)	
DOD (32/25)	10 (10%)	
DTC (1/1)	3 (3%)	
DUC/DOC (31/28)	10 (10%)	
NA	14	

NED - no evidence of disease, AWD - alive with disease, DOD - death of disease, DTC - death of treatment complication, DUC - death of uncertain cause, DOC - death of other cause, FU – follow-up, NA - data not available, OCCC – primary ovarian clear cell carcinoma. Percentage is counted only from available data and are rounded up/down.

### Next generation sequencing (NGS) analyses

The isolation of nucleic acids from FFPE tumor tissue for further DNA NGS and RNA-Seq analyses was performed as described previously [25]. Out of the 120 cases included into the study, 113 OCCCs were eligible for DNA and/or RNA NGS. Samples insufficient for complex molecular analyses were excluded (20/120 DNAs; 17%, and 15/120 RNAs; 13%, respectively), 92 samples had both complete DNA and RNA NGS analyses.

### DNA NGS analysis

Sequence capture NGS analysis of DNA was performed for 100 qualitatively sufficient OCCC cases in order to assess mutation pattern and frequency, tumor mutation burden (TMB), and microsatellite instability (MSI). Copy number variation (CNV) analyses were not performed because of the low DNA quality not suitable for reliable CNV testing.

The library preparation was performed using the KAPA HyperPlus kit [according to KAPA HyperCap Workflow v3.0 (Roche, Basel, Switzerland)] and a panel of hybridization probes against multiple targets of cancer relevant genes (727 genes or gene parts; 2097 kbp of the target sequence including 1708 kbp of coding regions; Roche; Supplementary Information). The prepared sample libraries were pair-end sequenced by the NextSeq 500 instrument (Illumina, San Diego, California) using the NextSeq 500/550 High Output Kit v2.5 (Illumina). The biostatistical evaluation using NextGENe software (Softgenetics) and the interpretation of DNA variants was performed as follows.

All the frameshift, no-start, and no-stop splice variants in the consensus splice sites, nonsense variants, and missense variants known as pathogenic and/or likely pathogenic (class 4/5 mutations; except for one nonsense *POLE* mutation which does not cause ultramutated phenotype) according to the ClinVar database were considered as deleterious. Detailed pipelines of all the NGS data analysis together with the module settings are available upon request. The analysis does not allow for the distinction between somatic and germline variants. The *TP53* variants were classified according to https://p53.iarc.fr/, ClinVar (https://www.ncbi.nlm.nih.gov/clinvar/), and https://www.cancerhotspots.org/. The size of the sequenced panel enabled us to also assess the TMB (number of mutations per 1 Mega base; mut/Mb); however, TMB was calculated only for samples with ≥ 40% tumor cells. Samples with TMB ≥ 10 mut/Mb were considered TMB-High. All synonymous and nonsynonymous variants with an allele frequency of ≥ 10% were counted. Furthermore, potential germline variants [according to databases of known germline polymorphisms including the Single Nucleotide Polymorphism database (dbSNP) and Exome Aggregation Consortium (ExAC)] and known or probable driver mutations (according to the COSMIC and ClinVar database) were determined. The resulting mutation number was normalized to 1 Mb. MSI was evaluated from NGS data using CLC Genomics Workbench software (CLC GW; Qiagen, Venlo, The Netherlands) and a module ‘Detect MSI status’ with default settings. When more than 20% of the 17 evaluated microsatellite markers were unstable, then the sample was considered microsatellite unstable (detailed analysis of the microsatellite status including a comparison of its assessment using several methods will be part of a forthcoming study). We also evaluated the hot spot variants in the *TERT* promoter (*TERT*p) that are clinically relevant (c.-124 C > T rs1561215364, c.-124 C > A, and c.–146 C > T rs1561215364).

### RNA NGS analysis

Total RNA samples were processed according to the KAPA RNA HyperPrep Kit protocol (Roche; input 300 ng where available; denaturation/fragmentation 85 °C – 2 min; 11 cycles of PCR). In those samples of sufficient quality (n = 105), the target sequences were enriched by the standard KAPA HyperCap Workflow v3 (Roche) using a custom panel focused on the pan-cancer markers and potential fusion genes (147 genes; 373 kbp of the target DNA sequence; Roche; Supplementary Information). The final libraries were pair-end sequenced by the NextSeq 500 instrument using 300 cycles chemistry kits (Illumina) with a target of 10 million single reads.

The sequencing data were analyzed using the CLC GW v21.0.5. (Qiagen) by an in-house pipeline which includes targeted RNA-Seq expression analysis (RNA-Seq Analysis module) and detection of fusion genes (Detect and Refine Fusion Genes module). The bioinformatics pipeline and module settings are available upon request.

All fusions identified by the CLC GW were manually checked, filtered, and confirmed using IGV v2.11.3. (Broad Institute, Cambridge, Massachusetts). Only those fusions meeting the following criteria were considered as true fusions: (i) fusions involving protein-coding genes with standard exon-exon junctions (± 15 bp range from exon boundary), with substantial expression when compared to other samples at the respective region, (ii) ≥ 10% of reads supporting fusion presence (crossing reads) out of read counts at the respective location. Frequently repeated fusions, fusions of genes from the same gene family, or transcriptional readthroughs were excluded and considered as artefacts. The nomenclature of the detected mutations follows HGVS recommendations (https://varnomen.hgvs.org/). Each fusion that was not described in the Quiver database (http://quiver.archerdx.com/), Fusion GDB (https://ccsm.uth.edu/FusionGDB/index.html), Mitelmandatabase (https://mitelmandatabase.isb-cgc.org/) or literature was considered as novel. OncoKB (https://www.oncokb.org/) was searched for the clinical significance of the detected fusions with respect to therapeutic actionability.

Expression profiling was performed using the Heat Map for RNA-Seq module in CLC GW with Manhattan distance and Complete linkage settings.

Hierarchical clustering based on the two-dimensional heat map of normalized expression values (RPKM) of all 147 gene targets in the panel was performed using the Heat Map for RNA-Seq module in CLC GW with Manhattan distance and Complete linkage settings.

Normalization of mRNA expression was evaluated as RPKM (reads per kilobase of transcript per million reads mapped) and *VCP*, *SF3B1*, and *ATP5F1B* genes were used as a reference.

### Statistical analysis

The software R (version 4.0.2, https://www.r-project.org/) was used to perform the statistical analyses.

Group comparisons were performed for categorical variables using Pearson chi-squared test or Fisher Exact test, based on excepted values.

Time-to-event analyses were computed using the package ‘survival’ and ‘survminer’. The associations between patient characteristics and survival were evaluated using univariable and multivariable Cox proportional hazard models (Cox PH) and described by the regression coefficient (β), hazard ratio (HR) with 95% confidence intervals (CI) and statistical significance. *P*-values were obtained by applying Wald statistics based on Cox PH model. Variables that showed significance in univariable model were selected for the multivariable Cox PH full model. A backward stepwise elimination was used to reach minimal adequate model. Survival curves were constructed by the Kaplan-Meier method based on the stratified risk score and compared by means of the log-rank test and likelihood ratio (LR) test.

Recurrence-free survival (RFS) was defined as the period from the date of diagnosis to the date of recurrence or death of disease, overall survival (OS) was defined as the period from the date of diagnosis to the date of recorded death. The length of the follow-up period (FU) was calculated from the date of diagnosis to the last recorded follow-up visit or death of patient. Among 87 patients included in the analysis, 21 died (24%), 10 of them from other cause than disease related.

All tests were two-sided and *p*-values < 0.05 were considered significant. Hierarchical clustering is based on the TMM method (trimmed mean of M values) expression data normalization, with additional calculation of TMM-adjusted log CPM (Counts per Million) for each gene with a subsequent Z-score normalization across all samples for each gene (counts for each gene are mean centered and scaled to unit variance).

Differential expression in two groups module, which is implemented in CLC GW, was used for the analysis of differential expression of individual genes included in panel between samples hierarchically clustered in group 2 versus group 1. This module is a multi-factorial statistics test based on a negative binomial Generalized Linear Model (the statistical model for this module is thoroughly described in the CLC GW manual - https://digitalinsights.qiagen.com/technical-support/manuals/).

## Results

### Genomic DNA alterations

**Fig. 1:**
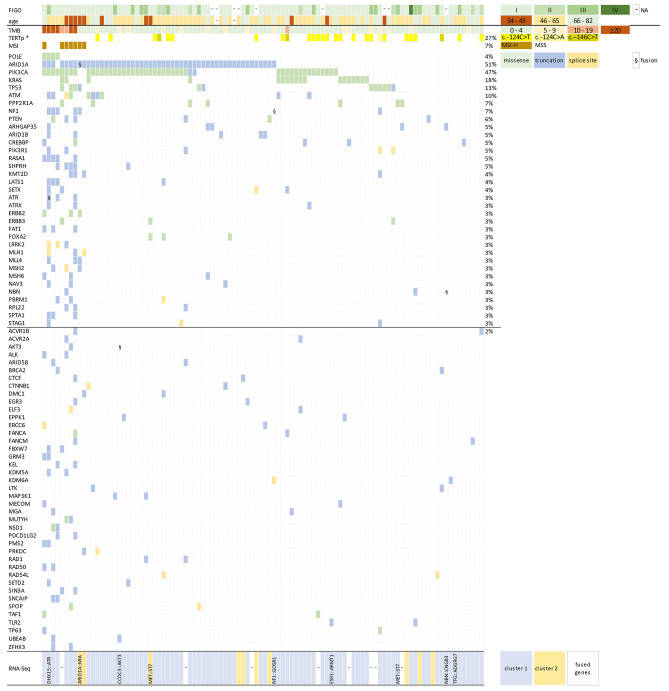
Molecular characteristics of 100 primary OCCCs analyzed by capture DNA sequencing of 527 genes. 75 genes with mutations detected in at least two cases are included. The whole spectrum of genes with class 4/5 mutations is provided in Supplementary Table 2

In total, the DNA NGS analysis of 100 eligible OCCC cases revealed pathogenic or likely pathogenic (class 4/5) mutations in 218 of the 727 analyzed genes (Supplementary Table 2). Out of those, for 144 genes class 4/5 mutation was detected only once in the whole sample set. The mean and median number of the detected somatic class 4/5 mutations per sample was 5.4 and 3, respectively (range 0–58). The spectrum and frequency of the mutated genes (only those affected in at least 2% of cases) in the context of clinicopathological variables and expression and fusion analysis is shown in Fig. 1. Table of genes with class 4/5 mutations in respective cases is provided in Supplementary Table 2.

The most frequently altered genes by class 4/5 mutations were *ARID1A* in 52/100 (51%), *PIK3CA* in 47/100 (47%), *TERTp* (27/100; 27%), *KRAS* 18/101 (18%), *TP53* 13/100 (13%), and *ATM* 10/100 (10%) cases. 4/100 (4%) cases showed *POLE* mutation, and 7/100 (7%) were MSI-High cases (one of which was also *POLE*^mut^).

The median tumor mutation burden for all 100 OCCCs was 3 mutations/megabase (TMB = 3 mut/Mb; range 0–86). Nine TMB-High OCCCs had median TMB = 25 mut/Mb (TMB range 10–86), of which 4 were *POLE*^mut^ (TMB range 24–86), 4 microsatellite instable (MSI-High; TMB range 14–26), and one sample was microsatellite stable (MSS) and without *POLE* mutation (*POLE*^*wt*^).

We correlated the mutation status in frequently mutated genes with clinico-pathological characteristics (Supplementary Table 3). In our data set we observed that mutations in *ARID1A* or in *PIK3CA* correlated with a younger age of patients (*p* = 0.043 or *p* = 0.002, respectively). The same was observed for group of *POLE*^mut^ and/or MSI-High patients compared with patients with *POLE*^wt^ and MSS (*p* = 0.015). The *KRAS* mutations were more common in the early stages of the disease (*p* = 0.020). The *KRAS* and *TP53* mutations were mutually exclusive (see Fig. 1).

### RNA-Seq – fusions transcript analyses

The RNA-Seq was successful in 105 cases. Only fusions involving different protein-coding genes in ≥ 10% of reads were reported. 13 different gene fusions were identified in 14/105 (13%) of the OCCCs. Of those, six were novel (*LAMB1*::*MET*, *CCDC3*::*AKT3*, *NBN*::*CNGB3*, *DHX15*::*ATR*, *LGALS3*::*EZH2*, *ARID1A*::*MIA2*) while seven have already been described in the databases or literature (Table 2). Two fusions were recurrent, namely *TFG*::*ADGRG7* (2/105; 2%), and *MET* fusions with different fusion partners (4/105; 4%). *MET* fusions included *ST7*::*MET* (2/105; each with different breakpoints), *CAPZA2*::*MET*, and *LAMB1*::*MET* (for fusion details see Supplementary Fig. 1 and Table 3). The 14 tumors with detected fusions did not show any specific morphologic parameters which would distinguish them from those without fusions (data not shown).

**Table 2:** The frequency of mutations in selected genes in 100 primary ovarian clear cell carcinomas compared to the literature

	Primary OCCCs		Literature OCCCs		
Gene	No. of cases Mut/all	Mutation %	No. of cases Mut/all	Mutation %	Range %
POLE	4/100	4	2/545	0	0–14
ARID1A*	51/100	51	678/1215	56	14-88
PIK3CA*	47/100	47	786/1825	43	13–67
KRAS	18/100	18	126/1169	11	0–43
TP53	12/100	12	208/1456	14	0–52
ATM	10/100	10	74/907	8	0–18
PPP2R1A	7/100	7	64/567	11	0–25
PTEN	6/100	6	47/866	5	0–17

*several cases with two alterations in the respective gene. mut - class 4/5 mutation, e.g. likely pathogenic/pathogenic mutation(s) detected; list of referred literature is in Supplementary Table 21

**Table 3:** Characterization of gene fusions detected among 105 OCCCs.

Fusion	Fusion (number of fusion reads/all reads from 5’gene)	Reading frame	chromosomes	strand	exon-exon boundary†	references
LAMB1::MET	LAMB1[NM_002291.3]:r.1_560_**MET**[NM_001127500.3]:r.3181_6876 (3729/6307)	IF	7;7	-;+	e5-e13	novel
CCDC3::AKT3	CCDC3[NM_031455.4]:r.1_692_AKT3[NM_005465.7]:r.363_7281 (112/602)	FS	10;1	-;-	e2-e3	novel
CAPZA2::MET	CAPZA2[NM_006136.3]:r.1_180_**MET**[NM_001127500.3]:r.1789_6876 (1093/3793)	FS	7;7	+;+	e3-e4	yes
LGALS3::EZH2	LGALS3[NM_002306.4]:r.1_650_EZH2[NM_004456.4]:r.440_2723 (33/47)	IF	14;7	+;-	e5-e4	novel
ST7::MET	ST7[NM_021908.3]:r.1_191_**MET**[NM_001127500.3]:r.1597_6876 (56/766)	FS	7;7	+;+	e1-e3	yes*
ST7::MET	ST7[NM_021908.3]:r.1_191_**MET**[NM_001127500.3]:r.3034_6876 (514/4312)	FS	7;7	+;+	e1-e12	yes*
ARID1A::MIA2	ARID1A[NM_006015.6]:r.1_1526_MIA2[NM_005930.4]:r.139_3630 (22/260)	IF	1;14	+;+	e1-e2	novel
DHX15::ATR	DHX15[NM_001358.3]:r.1_1646_ATR[NM_001184.4]:r.7071_8158 (42/588)	IF	4;3	-;-	e8-e42	novel
ERBB4::IKZF2	ERBB4[NM_005235.3]:r.1_354_IKZF2[NM_016260.3]:r.412_9515 (258/641)	IF	2;2	-;-	e1-e4	yes
NBN::CNGB3	NBN[NM_002485.4]:r.1_1234_CNGB3[NM_019098.4]:r.387_4347 (66/71)	IF	14;7	-;-	e9-e4	novel
ESR1::ARMT1	ESR1[NM_001122742.1]:r.1_1466_ARMT1[NM_024573.3]:r.122_2397 (51/305)	FS	14;7	+;+	e6-e2/3	yes
NF1::GOSR1	NF1[NM_000267.3]:r.1_443_GOSR1[NM_004871.3]:r.179_5993 (942/20)	FS	17;17	+;+	e1-e3	novel
TFG::ADGRG7 (2x)	TFG[NM_001007565.2]:r.1_453_ADGRG7[NM_032787.3]:r.372_3128	IF	3;3	+;+	e3-e2	yes

Fusion analysis was based on capture RNA-Seq data (147 gene panel) analysed by CLC Genomic workbench (Qiagen). Chr - chromosome (GRCh38/hg38), IF – predicted inframe fusion, FS – predicted frameshift fusion, Strand – location of 5’- and 3’-genes, exon-exon - predicted exon-exon boundary of 5’- and 3’-genes. As novel were considered fusion that were not described in Quiver database (http://quiver.archerdx.com/), Fusion GDB (https://ccsm.uth.edu/FusionGDB/index.html), Mitelmandatabase (https://mitelmandatabase.isb-cgc.org/) or literature. * OncoKB = likely oncogenic, † selected fusions confirmed by Sanger sequencing are underlined. A representative electropherogram confirming breakpoints for fusion *ST7*[NM_021908.3]:r.1_191_*MET*[NM_001127500.3]:r.3034_6876 is in Supplementary Fig. 1

9/92 cases with complete DNA and RNA analysis had a gene fusion detected (Fig. 1). Out of those, 6 had a concurrent aberration in at least one of the considered drivers *ARID1A*, *PIK3CA*, or *KRAS*. One case with MET::ST7 fusion had a concurrent mutation in *PPP2R1A*, one case with fusion *NBN*::*CNGB3* had a concurrent mutation in *ARHGAP35*, and one case with *TGF*::*ADGRG7* fusion had a concurrent mutation in *KDM5C*. None of the nine cases with a gene fusion had a concurrent hot-spot alteration in the *TERT* promoter.

### RNA-Seq – expression profile

**Fig. 2:**
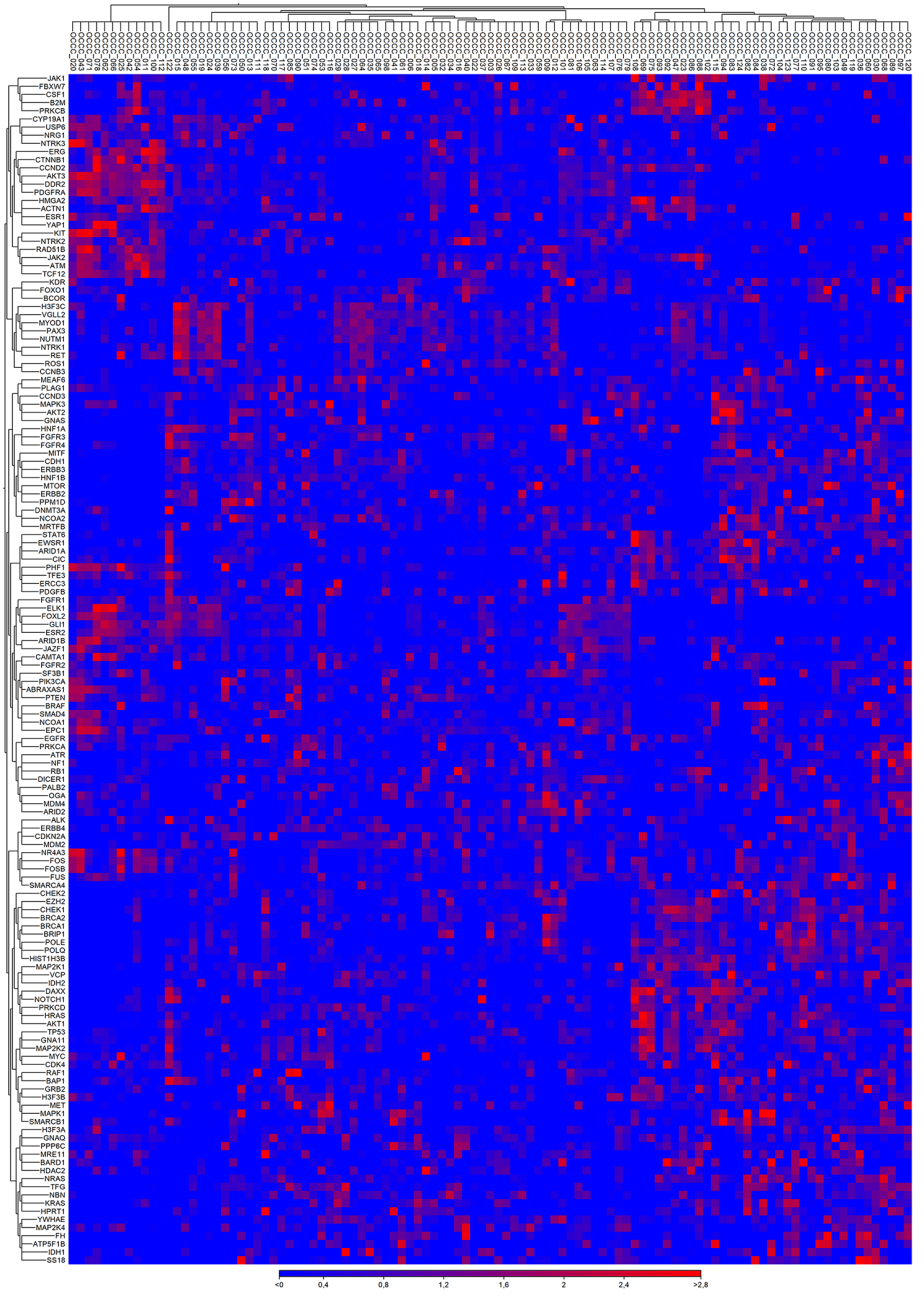
Visualization of hierarchical clustering based on the expression profiles of 105 OCCCs analyzed by 147 gene panel using capture RNA-seq. Hierarchical clustering was performed using Heat Map for RNA-Seq Analysis module in CLC Genomics Workbench v21.0.5. (CLC GW; Qiagen) with Manhattan distance and Complete linkage settings

The expression analysis of 147 genes was possible in 105 cases and revealed a heterogenous expression pattern. Unsupervised hierarchical clustering suggested two main clusters (Fig. 2). The detailed analysis of additionally normalized expression data to reference genes (*VCP*, *SF3B1*, *ATP51B*) comparing cluster 1 (93 cases) with cluster 2 (12 cases) is in Supplementary Table 5. On the mRNA level cluster 2 shows higher expression (≥ 2fold change) of *AKT3*, *DDR2*, *CTNNB1*, *JAK2*, *KIT* or *PDGFRA* in a majority of the samples and a lower expression of *CDH1*, *ERBB2*, *ERBB3*, *FGFR3*, *HIST1H3B*, *HNF1B* and *POLQ* in all samples when compared with cluster 1 (p < 0.00001).

Concerning the morphological features, cluster 2 was associated with macronucleoli (*p* = 0.031), increased mitotic activity (*p* = 0.014), and less necrosis (p < 0.001; Supplementary Table 4). We did not find any significant correlations of the expression pattern with regards to age, FIGO, or patient outcomes (Supplementary Table 3).

### Survival analyses

The follow-up data was available for 87 cases out of the 100 OCCCs. The median follow-up period was 3 years, mean ± SD was 3.5 ± 0.3 years. Cases which were *POLE*^mut^ and/or MSI-High (n = 9) had median follow-up 42 months, while compared with median 33 months in cases with *POLE*^wt^ and/or MSS. Based on univariate analysis, *POLE*^mut^ and/or MSI-High cases had favorable RFS (p = 0.04; no case with *POLE*^mut^ or MSI-High had an event compared with *POLE*^wt^/MSS cases). However, multivariable model adding age, tumor stage, radicality of surgical resection, and adjuvant therapy into consideration showed only the radicality of surgical resection as significant prognostic marker, indicating that cases with reached R0 resection margin correlated with strong decreased risk of relapse. No other stratification into subgroups based on the molecular features, including classification defined by TCGA for endometrial carcinoma (*POLE*^mut^, MSI-High, p53abn, and NSMP), classification based on the presence of *ARID1A*, *PIK3CA*, *TERT*, *TP53* or *KRAS* mutation, and classification based on expression profiling, showed prognostic significance.

## Discussion

The molecular features of OCCC can be of prognostic and predictive significance, but the current literary data is limited and equivocal. The results of previous studies showed a wide range of mutation frequency of several genes. An explanation for these differences could include the small sample sets in some previous studies, differences in methodology, or bias caused by the inclusion of tumors of other histogenesis, especially EC and HGSC with clear cell change. In our study, only tumors meeting strict diagnostic criteria were included, which prevented bias caused by the inclusion of tumors of other histogenesis. We revealed 18 recurrently mutated targets (detected in ≥ 4 patients), namely *ARID1A* (52/100; 52%); *PIK3CA* (47/100; 47%); *TERT* promoter hot-spot mutations (27/100; 27%); *KRAS* (18/100; 18%; with one case carrying the G12C mutation); *TP53* (13/100; 13%); *ATM* (10/100; 10%); *PPP2R1A* (7/100; 7%); *NF1*, *PTEN* (6/100; 6%); *ARHGAP35*, *ARID1B*, *CREBBP*, *PIK3R1*, *RASA1*, *SHPRH* (5/100; 5%); *LATS1*, *MLL2*, *POLE*, *SETX* (4/100; 4%). The majority of altered genes code for proteins which are involved in the PI3K/AKT and/or RAS/MAPK signaling pathways (*KRAS*, *NF1*, *PIK3CA*, *PIK3R1*, *PTEN*, *RASA1*), DNA repair and cell cycle regulation (*ATM*, *LATS1*, *MLL2*, *POLE*, *PPP2R1A*, *SETX*, *SHPRH*, *TP53*), and/or chromatin remodeling (*ARID1A*, *ARID1B*, *CREBBP*, *KMT2D*). Interestingly, in our study on Caucasian patients we did not detect any *BRAF* mutation, compared to 2/48 (4%) described in one study performed on the Japanese population [6]. However, our finding is in concordance with the results of others [3, 4].

We identified the *POLE* mutation in 4/100 (4%) of cases, which is discordant to the previous 9 studies analyzing *POLE* mutations in OCCC (Supplementary Table 1), in which *POLE* mutation was detected in only 2 of 405 cases. Of note, another case in our sample set carried a nonsense *POLE* mutation NM_006231.2:c.3961 A > T, p.(R1321X) which is currently considered as a variant of uncertain significance (VUS). In one study, the authors classified the tumors into TCGA-based molecular subtypes, including *POLE*^mut^, MMR-D, p53abn, and NSMP. When this approach is used in our study, the results are similar for NSMP tumors (76% in both ours and their cohort), but different for other subtypes (*POLE*^mut^ 4% in our study vs. 0.9% in theirs, MMR-D 7% in our study vs. 3.5%, and p53abn 13% in our study vs. 20%) [12]. In our study, OCCC cases with *POLE*^mut^ and/or MSI-High status had a better prognosis (favorable RFS, *p* = 0.041) compared to *POLE*^wt^ /MSS primary OCCC cases using univariate analysis, which is in concordance with recently published data [12]. However, the prognostic significance of this molecular trait was not confirmed in multivariable analysis in the context of other prognostic factors (age, stage at diagnosis, reaching R0 surgical treatment, and adjuvant therapy) where strongest prognostic factor was R0 resection margin.

Unlike the results reported in two previous studies, we did not observe worse survival in *TP53* mutated OCCC (our study showed a trend towards favorable RFS and OS for *TP53*^mut^ cases compared to other cases).

Another possible prognostic molecular marker suggested by one study is a mutation in the promoter region of *TERT* [26]. However, in our study the presence of *TERT*p hot-spot mutations was not associated with worse survival.

The co-occurrence of mutations in *ARID1A, PIK3CA, PIK3R1*, and *KRAS* was common among primary OCCC. *KRAS* and *TP53* mutations in our study were mutually exclusive (except for one MSI-High tumor). Our results are in disagreement with another study in which mutual exclusivity between somatic mutations of *ARID1A*, *TP53*, *PIK3CA*, and *PIK3R1* was found. In this study statistically significant co-occurrence between mutations in *ARID1A*, *PIK3CA*, *or TP53* and *BRCA1/BRCA2* was also found [3]. In our cohort, *BRCA1* (1/100) or *BRCA2* (2/100) mutations were very rare. Such discrepancies could be once again explained by the inclusion of cases of other histogenesis, as acknowledged by the authors, such as HGSC with clear cell change, which would also explain the higher frequency of *TP53* mutations in their study [3].

In our study, 20/100 cases (20%) showed no mutation in any of the commonly mutated genes, including *ARID1A*, *PIK3CA*, *TERT*, *KRAS*, *TP53*, *ATM*, and *NF1*. RNA analysis of these cases showed gene fusion in 3/19 cases (16%). Of the other cases which did show mutation in some of the commonly mutated genes, gene fusion was detected less frequently (6/73; 8% cases). This OCCC group carried none or a maximum of three genomic alterations (mainly involved in DNA repair, chromatin remodeling, or PI3K/AKT signaling). Altogether, we found gene fusions in 14/105 cases. A majority of fusions were intrachromosomal rearrangements, only 4/13 resulted from interchromosomal translocation. In 5/14 (36%) cases, the 3´ gene partner was a tyrosine kinase receptor. This finding can be of clinical significance, given that several tyrosine kinase inhibitors are currently available in clinical practice. In total, we have found *MET* fusions in 4/105 (4%) OCCCs. Two of them were *ST7*::*MET* fusions which have been previously reported in 1 HGSC and 1 OCCC, each with different breakpoints, which according to the database OncoKB [ [27]; accessed September 30, 2022] were considered to be likely oncogenic [15]. Another fusion, *CAPZA2*::*MET* has also been previously described in the Mitelman database of Chromosome Aberrations and Gene Fusions in Cancer (https://mitelmandatabase.isb-cgc.org; accessed 16, Oct 2022). Finally, we identified one novel *LAMB1*::*MET* fusion. The tyrosine kinase domain of MET was preserved in all reported fusions. Interestingly, a substantial part of the fusions affect the tyrosine kinase receptor molecule (6/13; *MET* fusions; *ERBB4*::*IKZF2* and *CCDC3*::*AKT3*). Novel fusions *NBN*::*CNGB3* and *DHX15*::*ATR* affect genes involved in DNA repair, *ESR1*::*ARMT1* affects genes involved in DNA damage response and PI3K/AKT signaling regulation.

The recurrent fusion *TFG*::*ADGRG7* has been reported previously in many malignancies (https://mitelmandatabase.isb-cgc.org; accessed 16, Oct 2022) and even in normal tissues [28].

The mRNA expression pattern was heterogeneous, but unsupervised hierarchical clustering analysis revealed two main clusters. Cluster 1 included 93 cases with variable expression pattern, while cluster 2 included 12 cases characterized by higher expression of *AKT3*, *DDR2*, *CTNNB1*, *JAK2*, *KIT* or *PDGFRA*, and lower expression of *CDH1*, *ERBB2*, *ERBB3*, *FGFR3*, *HIST1H3B* (also known as *H3C2*), *HNF1B* and *POLQ* when compared with cluster 1 (p < 0.001). Our results are not comparable with other studies focusing on expression profiling due to the very limited overlap in genes analyzed [3, 17].

Currently, there are no targeted therapies specific for OCCC, although a number of candidate targets have recently been identified and reviewed, including targets in PI3K/AKT/mTOR pathway, and ARID1A deficiency [29, 30]. Nevertheless, some molecular aberrations such as *NTRK* fusions, microsatellite instability, and high tumor mutation burden have predictive value in all solid tumors. In our study, the vast majority of altered genes are involved in PI3K/AKT and/or RAS/MAPK signaling pathways, DNA repair and cell cycle regulation, chromatin remodeling, or cell adhesion. The *ARID1A* mutated tumors could be potentially targetable by PARP inhibitors or HDAC inhibitors [31–34]. The presence of the *PIK3CA* mutation might indicate susceptibility to PI3K- or mTOR-inhibitors. Targets affecting the mTOR pathway have been detected in OCCC (including *PIK3CA*, *PTEN*, *FBXW7*, *PIK3R1*, *AKT3*, *NF1*). TMB-High (detected in 9% of cases) tumors may be eligible for immune therapy. The *BRCA1* and *BRCA2* mutations were very rare events, as well as MSI-High cases; however, those patients could be considered for treatment by PARP inhibitors or immunotherapy. Importantly, we identified frequently altered genes coding tyrosine kinase receptors, such as *MET*, *AKT3*, *DDR2*, *CTNNB1*, *KIT*, or *PDGFRA*, which are known therapeutic targets [35]. The MET inhibitors are currently considered as predictive biomarker in patients with lung adenocarcinoma [36] and c-MET has been considered as a potential therapeutic target in OCCC [27]. Another potential target could also be the overexpression of non-receptor tyrosine kinase JAK2 [37].

We are aware of the limitations of our study. The main limitation is that CNV and epigenetic changes were not performed. Moreover, analysis of the outcomes can be influenced by a limited number of events in our cohort.

## Conclusions

Knowledge about the molecular landscape of OCCC is gaining significance due to the expanding possibilities of targeted treatments available for several tumors. Our study described for the first time a complex molecular pattern in a pathologically well-defined sample set of 113 OCCC fulfilling strict inclusion criteria. Our results confirmed the favorable outcomes of *POLE*^mut^ and MSI-High OCCC. Moreover, the molecular landscape of OCCC revealed several potential therapeutical targets including *MET* fusion, and molecular testing can provide the potential for targeted therapy in patients with recurrent or metastatic tumors.

## Electronic supplementary material

Below is the link to the electronic supplementary material.

Supplementary Material 1

Supplementary Material 2

Supplementary Material 3

Supplementary Material 4

## Data Availability

All data supporting the findings of this study are available within the paper and its Supplementary Information. The datasets used and/or analyzed during the current study are available from the corresponding author upon reasonable request.

## List of abbreviations

OCCC: Ovarian clear cell carcinoma
EC: Endometrioid carcinoma
HGSC: High grade serous carcinoma
LGSC: Low grade serous carcinoma
MC: Mucinous carcinoma
OS: Overall survival
FFPE: Formalin-fixed and paraffin-embedded
TMAs: Tissue microarrays
RFS: Relapse-free survival
LFS: Local recurrence-free survival
MFS: Metastasis-free survival
RPKM: Read per kilobase million
NSMP: No specific molecular profile
MMR-D: Mismatch repair deficient
class 4/5 mutation: Likely pathogenic or pathogenic mutation
