# Supplementary material for: A comprehensive molecular analysis of 113 primary ovarian clear cell carcinomas reveals common therapeutically significant aberrations
Source: Diagn Pathol. 2023 Jun 12;18:72. doi: 10.1186/s13000-023-01358-0 (PMC10259037; doi:10.1186/s13000-023-01358-0)
Supplement: Supplementary file 1 — Supplementary Material 1 [file 13000_2023_1358_MOESM1_ESM.docx]

**SUPPLEMENTARY INFORMATION**

**Gene panel used for DNA NGS analysis**

ABL1; ABL2; ABRAXAS2; ACVR1; ACVR1B; ACVR2A; ADGRA2; AIP; AJUBA; AKT1; AKT2; AKT3; ALK; ALOX12B; AMER1; ANKRD11; ANKRD26; APC; APEX1; AR; ARAF; ARFRP1; ARHGAP35; ARID1A; ARID1B; ARID2; ARID5B; ASXL1; ASXL2; ATM; ATMIN; ATR; ATRIP; ATRX; AURKA; AURKB; AXIN1; AXIN2; AXL; B2M; B4GALT3; BABAM1; BABAM2; BAP1; BARD1; BBC3; BCL10; BCL2; BCL2L1; BCL2L11; BCL2L2; BCL6; BCOR; BCORL1; BCR; BIRC3; BIRC5; BIRC5 p; BLM; BMPR1A; BRAF; BRAP; BRCA1; BRCA2; BRCC3; BRD4; BRIP1; BTG1; BTG2; BTK; BUB1B; EMSY; CALR; CARD11; CASP8; CBFB; CBL; CCDC6; CCND1; CCND2; CCND3; CCNE1; CD22; CD274; CD276; CD70; CD74; CD79A; CD79B; CDA; CDC73; CDH1; CDK12; CDK4; CDK6; CDK8; CDKN1A; CDKN1B; CDKN1C; CDKN2A; CDKN2B; CDKN2C; CEBPA; CENPA; CEP57; CFTR; CIC; CLSPN; CLTC; COP1; CREBBP; CRIPAK; CRKL; CRLF2; CSF1R; CSF3R; CSNK1A1; CSNK1D; CSNK1E; CTCF; CTLA4; CTNNA1; CTNNB1; CUL3; CUL4A; CUX1; CWF19L2; CXCR4; CYLD; CYP17A1; CYP19A1; DAXX; DCK; DCLRE1C; DCTD; DCUN1D1; DDB2; DDR1; DDR2; DDX41; DHFR; DHX15; DICER1; DIS3; DMC1; DNAJB1; DNAJC21; DNMT1; DNMT3A; DNMT3B; DOT1L; DPYD; E2F3; EED; EGFL7; EGFR; EGR3; EIF1AX; EIF4A2; EIF4E; ELF3; ELK1; ELOC; EML4; EP300; EPCAM; EPHA3; EPHA5; EPHA7; EPHB1; EPHB4; EPHB6; EPHX1; EPPK1; ERBB2; ERBB3; ERBB4; ERCC1; ERCC2; ERCC3; ERCC4; ERCC5; ERCC6; ERG; ERRFI1; ESR1; ESR2; ETS1; ETV1; ETV4; ETV5; ETV6; EWSR1; EXO1; EXT1; EXT2; EYA2; EZH2; F11R; FAAP24; FAM175A; FAM46C; FAN1; FANCA; FANCB; FANCC; FANCD2; FANCE; FANCF; FANCG; FANCI; FANCL; FANCM; FAS; FAT1; FBXW7; FGF1; FGF10; FGF12; FGF14; FGF19; FGF2; FGF23; FGF3; FGF4; FGF5; FGF6; FGF7; FGF8; FGF9; FGFR1; FGFR2; FGFR3; FGFR4; FH; FLCN; FLI1; FLT1; FLT3; FLT4; FOXA1; FOXA2; FOXL2; FOXO1; FOXP1; FRS2; FUBP1; FYN; GABRA6; GADD45A; GATA1; GATA2; GATA3; GATA4; GATA6; GEN1; GID4; GLI1; GNA11; GNA13; GNAQ; GNAS; GPC3; GPS2; GRB2; GRB7; GREM1; GRIN2A; GRM3; GSK3B; H2AX; H3F3A; H3F3B; H3F3C; HDAC1; HDAC2; HELQ; HGF; HIST1H1C; HIST1H2BD; HIST1H3A; HIST1H3B; HIST1H3C; HIST1H3D; HIST1H3E; HIST1H3F; HIST1H3G; HIST1H3H; HIST1H3I; HIST1H3J; HIST2H3A; HIST2H3C; HIST2H3D; HIST3H3; HLA-A; HLA-B; HLA-C; HMGA2; HNF1A; HNF1B; HNRNPK; HOXB13; HRAS; HSD3B1; HSP90AA1; HUS1; CHD2; CHD4; CHEK1; CHEK2; ICOSLG; ID3; IDH1; IDH2; IFNGR1; IGF1; IGF1R; IGF2; IKBKE; IKZF1; IL10; IL7R; INHA; INHBA; INPP4A; INPP4B; INSR; IRF2; IRF4; IRS1; IRS2; JAK1; JAK2; JAK3; JAM2; JAM3; JUN; KAT5; KAT6A; KCNJ5; KDM5A; KDM5C; KDM6A; KDR; KEAP1; KEL; KIF5B; KIT; KLF4; KLHL6; KMT2A; KMT2B; KMT2C; KMT2D; KRAS; LAMP1; LATS1; LATS2; LIFR; LIG1; LIG3; LIG4; LMO1; LRIG1; LRP1B; LRRK2; LTK; LYN; LZTR1; MAF; MAGI2; MALT1; MAP2K1; MAP2K2; MAP2K4; MAP3K1; MAP3K13; MAP3K14; MAP3K4; MAPK1; MAPK3; MAPK8IP1; MAX; MCL1; MCPH1; MDC1; MDM2; MDM4; MECOM; MED12; MEF2B; MEN1; MERTK; MET; MGA; MGMT; MITF; MKNK1; MLH1; MLH3; MLLT3; MMP8; MPL; MRE11A; MSH2; MSH3; MSH5; MSH6; MSR1; MST1; MST1R; MTAP; MTDH; MTOR; MUS81; MUTYH; MYB; MYC; MYCL; MYCN; MYD88; MYOD1; NAB2; NACC2; NAT1; NAV3; NBN; NCAM1; NCOA3; NCOA4; NCOR1; NEGR1; NELFB; NF1; NF2; NFE2L2; NFE2L3; NFKBIA; NFKBIZ; NHEJ1; NKX2-1; NKX3-1; NOTCH1; NOTCH2; NOTCH3; NOTCH4; NPM1; NRAS; NRG1; NSD1; NSD2; NSD3; NT5C2; NT5C2; NT5C3A; NT5C3B; NT5E; NTRK1; NTRK2; NTRK3; NUP93; NUTM1; OGG1; P2RY8; PAK1; PAK3; PAK7; PALB2; PARD3; PARP1; PARP2; PARP3; PAX3; PAX5; PAX7; PAX8; PBRM1; PCBP1; PCNA; PDCD1; PDCD1LG2; PDGFRA; PDGFRB; PDK1; PDPK1; PGR; PHB; PHF6; PHOX2B; PIK3C2B; PIK3C2G; PIK3C3; PIK3CA; PIK3CB; PIK3CD; PIK3CG; PIK3R1; PIK3R2; PIK3R3; PIM1; PLA2G2A; PLCG2; PLK2; PMAIP1; PMS1; PMS2; PNRC1; POLB; POLD1; POLE; POLQ; POT1; PPARA; PPARD; PPARG; PPM1D; PPP2R1A; PPP2R2A; PPP6C; PRDM1; PREX2; PRF1; PRKAR1A; PRKCI; PRKDC; PRKN; PRSS8; PRX; PTEN; PTCH1; PTPN11; PTPRD; PTPRO; PTPRS; PTPRT; PTTG2; QKI; RAB35; RAC1; RAD1; RAD17; RAD18; RAD21; RAD23B; RAD50; RAD51; RAD51AP1; RAD51B; RAD51C; RAD51D; RAD52; RAD54B; RAD54L; RAD9A; RAF1; RANBP2; RARA; RASA1; RB1; RBBP8; RBM10; RECQL; RECQL4; RECQL5; REL; RET; RFC1; RFC2; RFC4; RHBDF2; RHEB; RHOA; RICTOR; RIT1; RNF146; RNF168; RNF43; RNF8; ROS1; RPA1; RPL22; RPL5; RPS6KA4; RPS6KB1; RPS6KB2; RPTOR; RRM1; RUNX1; RUNX1T1; RYBP; SDHA; SDHAF2; SDHB; SDHC; SDHD; SETBP1; SETD2; SETX; SF3B1; SGK1; SH2B3; SH2D1A; SHPRH; SHQ1; SIN3A; SLC29A1; SLIT2; SLX4; SMAD2; SMAD3; SMAD4; SMARCA4; SMARCB1; SMARCD1; SMARCE1; SMC1A; SMC3; SMO; SNAI1; SNAI2; SNAI3; SNCAIP; SOCS1; SOX10; SOX17; SOX2; SOX9; SPEN; SPOP; SPTA1; SRC; SRSF2; STAG1; STAG2; STAT3; STAT4; STAT5A; STAT5B; STK11; STK40; SUFU; SUZ12; SYK; TAF1; TAZ; TBL1XR1; TBX3; TCF3; TCF7L2; TCL1A; TEK; TELO2; TERF2; TERT; TERT p; TET1; TET2; TFE3; TFRC; TGFBR1; TGFBR2; TIPARP; TJP1; TLR2; TLR4; TMEM127; TMPRSS2; TNFAIP3; TNFRSF14; TOP1; TOP2A; TOPBP1; TP53; TP53BP1; TP63; TPM3; TRAF2; TRAF7; TRRAP; TSC1; TSC2; TSHR; TSHZ2; TSHZ3; TSZH1; TWIST1; TWIST2; TYRO3; U2AF1; U2AF1 p; UBE2A; UBE2B; UBE2I; UBE2V2; UBE4B; UGT1A1; UIMC1; USP9X; VEGFA; VEZF1; VHL; VTCN1; WISP3; WRN; WT1; XIAP; XPA; XPC; XPO1; XRCC1; XRCC2; XRCC3; XRCC4; XRCC5; XRCC6; YAP1; YES1; ZBTB2; ZBTB7A; ZEB1; ZEB2; ZFHX3; ZNF217; ZNF350; ZNF365; ZNF703; ZRSR2

**Gene panel used for RNA NGS analysis**

ACTN1; AKT1; AKT2; AKT3; ALK; ARID1A; ARID1B; ARID2; ATM; ATP5F1B; ATR; B2M; BAP1; BARD1; BCOR; BRAF; BRCA1; BRCA2; BRIP1; CAMTA1; CCNB3; CCND2; CCND3; CDH1; CDK4; CDKN2A; CIC; CSF1; CTNNB1; CYP19A1; DAXX; DDR2; DICER1; DNMT3A; EGFR; ELK1; EPC1; ERBB2; ERBB3; ERBB4; ERCC3; ERG; ESR1; ESR2; EWSR1; EZH2; ABRAXAS1; FBXW7; FGFR1; FGFR2; FGFR3; FGFR4; FH; FOS; FOSB; FOXL2; FOXO1; FUS; GLI1; GNA11; GNAQ; GNAS; GRB2; H3F3A; H3F3B; H3F3C; HDAC2; HIST1H3B; HMGA2; HNF1A; HNF1B; HPRT1; HRAS; CHEK1; CHEK2; IDH1; IDH2; JAK1; JAK2; JAZF1; KDR; KIT; KRAS; MAP2K1; MAP2K2; MAP2K4; MAPK1; MAPK3; MDM2; MDM4; MEAF6; MET; OGA; MITF; MRTFB; MRE11; MTOR; MYC; MYOD1; NBN; NCOA1; NCOA2; NF1; NOTCH1; NR4A3; NRAS; NRG1; NTRK1; NTRK2; NTRK3; NUTM1; PALB2; PAX3; PDGFB; PDGFRA; PHF1; PIK3CA; PLAG1; POLE; POLQ; PPM1D; PPP6C; PRKCA; PRKCB; PRKCD; PTEN; RAD51B; RAF1; RB1; RET; ROS1; SF3B1; SMAD4; SMARCA4; SMARCB1; SS18; STAT6; TAF15; TCF12; TFE3; TFG; TP53; USP6; VCP; VGLL2; YAP1; YWHAE

**Areas with low or limited coverage**

In the DNA gene panel, there were targets with no coverage (including genes RANBP2 and FGF7; and selected exons in genes CHEK2_e13-15; PMS2_e2 + e4; HSP90AA1_e7 + e11; NPM_e11; RPS6KB_e13) or low coverage: AKT3_e9; APC_e5; AR_e1; ATM_e10; BCR_e19; BCR_e22; CLSPN_e22; CREBBP_e18; EIFE4_e7; EML4_e2; ERBB4_e8; GPC3_e3; H3-3A_e4; HELQ_e3; HLA-A_e4; HLA-B_e2; HLA-B_e3; HLA-B_e4; HLA-C_e3; HLA-C_e4; HNRNPK_e9; INSR_e3; KIT_e16; KMT2C_e38; MALT1_e3; MKNK1_e8; MLH1_e12; MMP8_e6; NF1_e13; NF1_e21; NF1_e24; NF1_e25; PMS2_e3; POLE_e25; POLQ_e18; POT1_e19; PRKCI_e1; PRKDC_e68; PTEN_e8; RAD21_e10; RAD21_e8; RECQL_e3; REL_e9; RPL_e6; RPL5_e5; SDHC_e5; SDHD_e4; SGK1_e4; SPEN_e9; STAG2_e11; STAG2_e16; STAG2_e21; TP53BP1_e13; TPM3_e3; TSC1_e21; ZEB1_e7).

These limitations disabled or lowered the sensitivity of detection of potential mutations in respective areas.

**Supplementary Figure 1. MET fusion transcripts.**

| A | 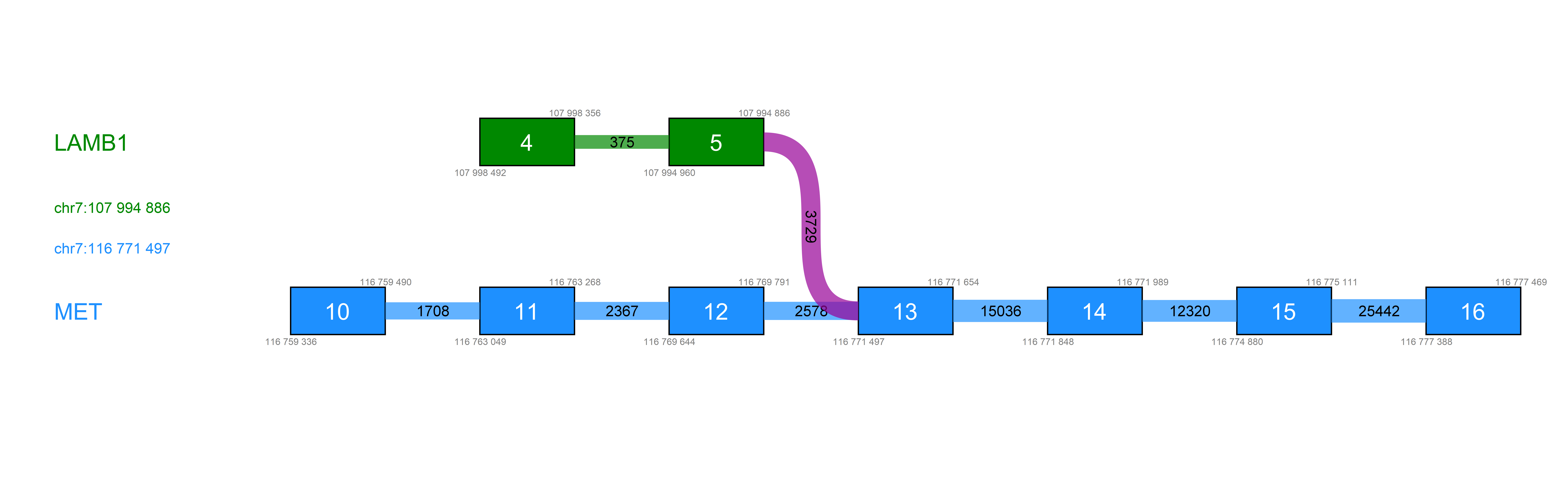 |
| --- | --- |
| B | 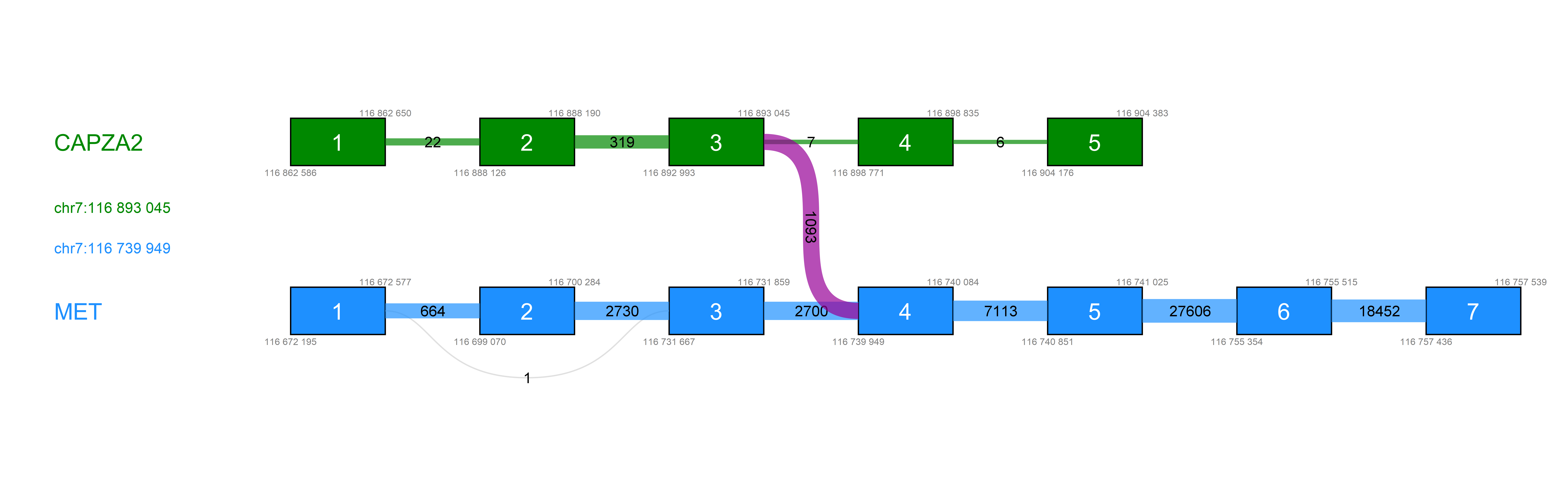 |
| C | 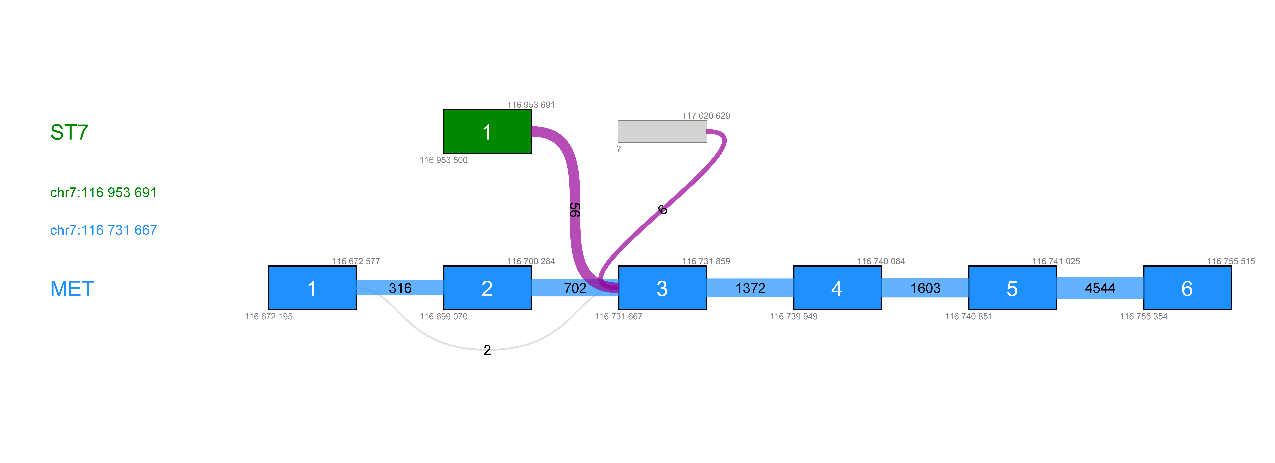 |
| D | 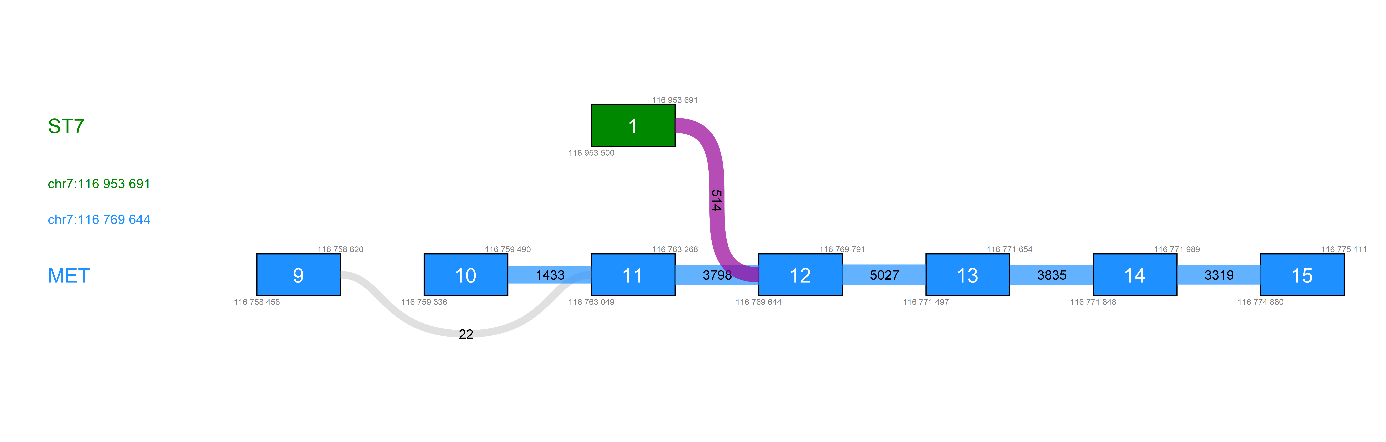 |
| E |  |
|  |  |
|  | Diagrams of four detected MET fusions (A-D) exported from CLC Genomics Workbench (Qiagen). Green – 5’-gene; blue – 3’-gene; blue/green boxes – numbered respective exons; blue/green lines – canonical exon-exon junctions with number of crossing reads; violet lines – fusion exon-exon junctions with a number of fusion crossing reads.  (E) A representative diagram of fused genes breakpoint analysis (diagram D; fusion ST7[NM_021908.3]:r.1_191_MET[NM_001127500.3]:r.3034_6876). Breakpoints verified by direct Sanger sequencing are indicated by a *dashed line*. The upper part of the diagram shows sequences upstream and downstream to the breakpoints. The highlighted sequences (yellow) correspond to the sequences in electropherogram. A 187 bp long junction fragment from complementary DNA was amplified using the primers 9F_ST7-MET: GACCGTGTGGTTCTTCATCGTGC, 9R_ST7-MET: GGGACCGTGCATAAAACGGCTTC.  and 5x HOT FIREPol® EvaGreen® qPCR Supermix (Solis Biodyne), and program: 95°C 15min, followed by 10 cycles (95°C 30s, 65°C to 55°C 50s, 72°C 30s), 25 cycles (95°C 30s, 40°C 50s, 72°C 30s), and 72°C 4min.  Reverse transcription was performed using 600 ng RNA and FusionPlex Reagents for Illumina - Random priming 2.0, First Strand cDNA Synthesis, and Second Strand cDNA Synthesis (ArcherDX) kit according to the manufacturer's instructions.  Direct Sanger sequencing was performed using BigDye v3.1 and the ABI3500 analyzer (TermoFisher). |

Supplementary Table 1. Literature review

Separate Excel file

Supplementary Table 2. A spectrum of genes with detected class 4/5 mutation in 100 primary OCCCs.

Separate Excel file

Supplementary Table 3. Correlation of mutation status with clinico-pathological characteristics on a set of 100 primary OCCCs where the DNA NGS data was available.

Supplementary Table 4. Morphological characteristics of 105 OCCCs and correlations with expression data.

|  |  | Expression analysis | | |
| --- | --- | --- | --- | --- |
| **Characteristic** | OCCC (N=105) | Cluster 1 (N=93) | Cluster 2 (N=12) | *p*-value |
| Macronucleolus |  |  |  | **0.031** |
| No (0%) | 7 (7%) | 4 | 3 |  |
| Yes (≥ 1%) | 98 (93%) | 89 | 9 |  |
| High grade atypia |  |  |  | 0.375 |
| No Absent | 48 (46%) | 41 | 7 |  |
| Yes Rare | 25 (24%) | 52* | 5* |  |
| Focal | 30 (28%) |  |  |  |
| Diffuse | 2 (2%) |  |  |  |
| Mitosis/10HPFs |  |  |  | **0.014** |
| No (0 mitosis/10HPF) | 10 (10%) | 6 | 4 |  |
| Yes (≥ 1 mitoses/10HPF) | 95 (90%) | 87 | 8 |  |
| range | 0-35 |  |  |  |
| mean/median | 5.8/4 |  |  |  |
| Necrosis |  |  |  | **<0.001** |
| No (0%) | 21 (20%) | 12 | 9 |  |
| Yes (≥ 1%) | 84 (80%) | 81 | 3 |  |
| PMN |  |  |  | 0.337 |
| No | 37 (35%) | 31 | 6 |  |
| Yes | 68 (65%) | 62 | 6 |  |
| Cluster 1 and 2 are subgroups resulting from the unsupervised hierarchical clustering of normalized expression data from panel RNA-Seq (see Figure 2), HPF - high-power fields, PMN - polymorphonuclear neutrophils, TILs - tumor-infiltrating lymphocytes. * Rare+focal+diffuse all together as a positive subgroup. All *p*-values are based on the Fisher Exact test. | | | | |

Supplementary Table 5. Normalized expression data to reference genes VCP, SF3B1, ATP51B

Separate Excel file.
